# Gestational Inulin Supplementation in Low-/High-Fat Sow Diets: Effects on Growth Performance, Lipid Metabolism, and Meat Quality of Offspring Pigs

**DOI:** 10.3390/foods14081314

**Published:** 2025-04-10

**Authors:** Pan Zhou, Yachao Wu, Jianbo Shen, Tao Duan, Long Che, Yong Zhang, Yang Zhao, Honglin Yan

**Affiliations:** 1 School of Life Science and Agro-Forestry, Southwest University of Science and Technology, 59 Qinglong Road, Mianyang 621010, China; pamelazhoupanpan@aliyun.com (P.Z.); yachaowu@163.com (Y.W.); 18981535212@163.com (J.S.); taoduan17@gmail.com (T.D.); zyzlrzjh@swust.edu.cn (Y.Z.); 2 College of Animal Science and Technology, Henan University of Animal Husbandry and Economy, No. 6 North Longzihu Road, Zhengdong New District, Zhengzhou 450046, China; chelong1989@126.com; 3 Animal Breeding and Genetics Key Laboratory of Sichuan Province, Sichuan Animal Science Academy, Chengdu 610066, China

**Keywords:** maternal nutrition, dietary fiber, epigenetics, lipid metabolism, meat quality

## Abstract

This study investigated whether the supplementation of prebiotic inulin to gestating sows programmatically affects offspring growth performance and meat quality while exploring its epigenetic effects through histone acetylation modulation. After mating, sixty multiparous sows (Landrace × Yorkshire; parity 2–3) were assigned to a 2 × 2 factorial arrangement with inulin (0% vs. 1.5%) and fat (0% or 5%) supplementation until farrowing. Post-weaning, five litters (10 piglets per litter) per treatment were selected and maintained in their original litter for fattening under standardized feeding. The results demonstrated that maternal inulin supplementation during gestation accomplished the following: (1) Increased offspring liver index by 13.4% at weaning and 6.8% at finishing (*p* < 0.05) while reducing the finishing-phase backfat thickness by 11.6% (*p* < 0.01), with a significant inulin × fat interaction attenuating fat-induced abdominal lipid accumulation at weaning (*p* = 0.05). (2) Decreased *longissimus dorsi* muscle lightness (L*) by 4.5% in finishing pigs (*p* = 0.02) without altering the other meat quality parameters. (3) Suppressed offspring liver lipid deposition at birth and finishing (*p* < 0.05), concomitant with upregulated hepatic *PGC-1α* and *CPT1A* expression (*p* < 0.05). (4) Elevated neonatal serum butyrate by 15.6% (*p* = 0.06) while inhibiting hepatic histone deacetylase (HDAC) activity and enhancing histone H3/H4 acetylation (*p* < 0.01). These findings suggest that maternal inulin supplementation during gestation mitigates offspring hepatic lipid deposition through butyrate-mediated epigenetic regulation, where microbial-derived butyrate from inulin fermentation inhibits HDAC activity, enhances histone acetylation levels, and upregulates fatty acid β-oxidation gene expression. This study provides novel mechanistic insights into how maternal dietary fiber nutrition programs offspring development through epigenetic reprogramming.

## 1. Introduction

Early life (fetal and lactation period) is considered a “critical window period” for individual growth and development. When the fetus is in the uterus, it is completely dependent on the maternal nutrient supply to maintain normal growth and development; thus, most environmental exposures are related to the maternal diet [1]. Compared to non-pregnant individuals, maternal nutrition during pregnancy affects not only the maternal metabolic state itself but also exerts lasting impacts on fetal developmental programming as well as postnatal metabolism, growth, and development [2,3]. Furthermore, part of these maternal environment-induced changes can be attributed to epigenetic processes [4,5]. Epigenetics refers to the regulation of gene expression through chromatin structure modifications that occur without altering the DNA sequence [6]. The main processes include DNA methylation, histone post-translational modifications, and non-coding RNAs [7]. In eukaryotic cells, genomic DNA is wrapped by histones into nucleosomes, which serve as the building blocks of chromatin [8]. Histones are small molecular basic proteins, and their modification generally occurs on the free amino termini of the four core histones (H2A, H2B, H3, and H4), including methylation/demethylation, acetylation/deacetylation, ubiquitination/deubiquitination, phosphorylation, SUMO (small ubiquitin-like modifier) modification, and biotinylation [9]. Histone acetylation is mediated by histone acetyltransferases (HATs) and histone deacetylases (HDACs) [10]. HATs can add acetyl groups to the amino termini of lysine residues, increasing the distance between DNA and histones, and thereby promoting the access of transcription factors to the promoter region of target genes. HDACs, in contrast, remove acetyl groups from the amino termini, preventing transcription factors from accessing chromatin regions [11]. The current investigations in livestock epigenetics are centered on two aspects: the progressive modulation of genomic epigenetic landscapes by environmental factors, which subsequently governs associated gene expression patterns, and the transgenerational inheritance mechanisms underlying both epigenetic signatures and their phenotypic manifestations [12]. Specifically, emerging evidence demonstrates the regulatory roles of epigenetic regulatory processes on livestock reproduction [13,14], growth and development [15,16], and health [17,18] in response to different nutritional stimuli.

Short-chain fatty acids (SCFAs), the metabolites of dietary fiber fermentation by microorganisms in the large intestine, are not only essential energy sources for the body but also critical cellular signaling molecules involved in a series of physiological and biochemical reactions [19]. Interestingly, they are also believed to regulate gene expression through epigenetic mechanisms [20]. Among them, butyrate, one of the end products, is known as the lowest-molecular-weight inhibitor of HDACs and is capable of regulating gene expression [21]. Butyrate molecule consists of a propyl group and a carboxyl group. The propyl group can bind to the active site of HDACs, thereby primarily acting as an inhibitor of class I and class II HDACs [22]. Currently, there are numerous studies investigating how butyrate regulates metabolism [23,24,25,26,27], inflammation responses [28,29,30,31], and immune responses [32,33,34,35] through histone deacetylation. However, research on the effects of maternal butyrate supplementation during pregnancy on the physiological metabolism and growth of their offspring has not been sufficiently explored. Without exception, all of these studies observed significant changes in offspring growth [36], embryo survival [37], lipid and energy metabolism [38,39,40,41], immune function [42], mitochondrial function [43], and neurocognitive function [44] when butyrate was supplemented to maternal diets during pregnancy or the pregnancy-lactation period. It is worth noting, however, that only a few studies have investigated the role of histone acetylation modifications in this context. Given that SCFAs serve as the primary microbial metabolites of dietary fiber with established epigenetic regulatory functions, we hypothesize that maternally derived SCFAs from dietary fiber fermentation undergo placental transfer to induce fetal epigenetic reprogramming through histone deacetylation, thereby programming postnatal growth and metabolic trajectories. Therefore, this study aims to investigate the effects of maternal inulin supplementation on offspring growth performance and lipid metabolism, examine the underlying epigenetic mechanisms mediated through histone acetylation modulation, and assess the persistence of these epigenetically driven phenotypic changes into adulthood with their consequent effects on meat quality parameters.

## 2. Materials and Methods

### 2.1. Animals and Experimental Design

After mating, sixty multiparous sows (Landrace × Yorkshire; parity 2–3) were assigned to a 2 × 2 factorial arrangement, involving two inulin levels (0% and 1.5%) and two fat levels (0% and 5%), based on parity, body weight, and backfat thickness. The four treatment groups were low-fat diet (LFD), low-fat diet with 1.5% inulin (LFD.Inu), high-fat diet (HFD), and high-fat diet with 1.5% inulin (HFD.Inu). Throughout the gestation period, the sows received their assigned experimental diets, whereas during the lactation all the sows were provided with a standardized lactation diet. After weaning, five litters (10 piglets per litter) whose average piglet weaning weight closely matched the treatment-specific mean values were selected from each treatment and maintained in their original litter for fattening. Therefore, in this study, the litter was considered the experimental unit, with five replicates (litters) per treatment group. During the experiment, standardized feed was offered to all the offspring pigs at each stage. The research protocol was approved by the Care and Use Committee of Sichuan Agricultural University under ethics approval number DKY-B20121601.

### 2.2. Diet and Feeding

All the diets for the sows were formulated to meet or exceed the nutrient requirements of gestating and lactating sows, as recommended by the NRC (2012). Detailed information about the diets can be found in Supplementary Table S1. Inulin used in the study was obtained from BENEO-Orafti (Orafti GR, Tienen, Belgium), and had a purity greater than 90% and average degree of polymerization between 10 and 12. The post-weaning diets for the offspring pigs were formulated in five stages: 28–42 days, 42–70 days, 70–110 days, 110–150 days, and 150–180 days. The ingredients and nutrient composition of these diets are presented in Table S2.

After weaning, the piglets were kept in the sow’s pens for one week, with the room temperature maintained above 25 °C. During this period, the weaned piglets were fed five times daily (8:00, 11:00, 14:00, 17:30, and 20:30) with ad libitum access to feed and water. At day 35 of age, the piglets were moved to nursery pens, where they were fed three times daily (8:00, 12:00, and 16:00) with free access to feed and water, and the room temperature was maintained at 20–22 °C. At day 70 of age, the pigs were transferred to fattening pens, where they were fed twice daily (8:00 and 16:00) with ad libitum access to feed and water, and the room temperature was maintained at 18–20 °C.

### 2.3. Sample Collection

#### 2.3.1. Collection of Blood Samples from Newborn Piglets

After birth (prior to colostrum ingestion), six male piglets per treatment with body weights close to the mean within-litter weight were selected for pre-suckling blood sampling. Blood was collected into two 5 mL tubes without anticoagulant and left at room temperature for 2 h, followed by centrifugation (2550× *g*, 10 min, 4 °C) according to Zhou et al. (2023) [45]. Serum samples were harvested and stored at −20 °C pending analysis.

#### 2.3.2. Collection of Tissue Samples from Newborn Piglets and Finishing Pigs

The newborn piglets selected for blood analysis were sacrificed. At day 180 of age, five finishing pigs per treatment, with body weights close to the litter average, were selected for slaughter after overnight fasting. Samples from the left major lobe of the liver (excluding the gallbladder) were collected, flash-frozen in liquid nitrogen, and stored at −80 °C for gene analysis. The liver samples (10 g from newborn piglets or 50 g from finishing pigs) were stored at −20 °C for lipid content analysis. Additionally, fresh liver sections were placed in a 4% paraformaldehyde solution for subsequent hematoxylin and eosin (HE) staining analysis.

### 2.4. Feed Intake and Body Weight Measurements at Different Stages of Pigs

Feed intake and feed residues were recorded daily per litter, and the average daily feed intake (ADFI) was calculated for each growth stage. The weaning weights of the piglets were recorded. Subsequently, individual pigs were weighed after fasting in the early morning at day 70, 110, 150, and 180 of age.

### 2.5. Concentration of Serum Volatile Fatty Acids in Newborn Piglets

The serum was analyzed for volatile fatty acids (VFAs, mainly acetate, propionate, and butyrate) using gas chromatography (Varian CP-3800 GC, VARIAN, Palo Alto, CA, USA) as described by Zhou et al. (2018) [46].

### 2.6. Hepatic Lipid Content and H&E Staining in Newborn Piglets and Finishing Pigs

The frozen liver samples were freeze-dried to constant weight, and then ground and mixed thoroughly to analyze for lipid content following AOAC (2007) [47]. The liver tissues were removed from the 4% paraformaldehyde solution, deparaffinized and stained with hematoxylin, and hydrated sequentially. Differentiation was then performed in a 1% hydrochloric acid solution with subsequent rehydration. The samples were then stained with eosin, dehydrated through an alcohol series, and mounted with neutral resin.

### 2.7. Determination of HDAC Activity and Histone Acetylation in the Liver of Newborn Piglets

The determination of acetylation of histones H3 and H4 in the liver mainly involved two steps: histone extraction and acetylated H3 and H4 quantification. The detailed protocol is as follows: (1) Histones were extracted from the liver tissue using the EpiQuik™ Total Histone Extraction Kit (Base Catalog #P-0006; Epigentek, Farmingdale, NY, USA) following the instruction of the manufacturer. (2) Histone H3 and H4 acetylation levels were measured using the EpiQuik™ Total Histone H3 Acetylation Detection Fast Kit (Colorimetric) (Base Catalog #P-4030; Epigentek, Farmingdale, NY, USA) and the EpiQuik™ Total Histone H4 Acetylation Detection Fast Kit (Colorimetric) (Base Catalog #P-4032; Epigentek, Farmingdale, NY, USA), respectively, according to the manufacturer’s protocol. The principle of the assay is that acetylated H3 or H4 is captured by coated anti-acetyl histone H3 or H4 antibodies, followed by detection with a labeled secondary antibody. Acetylation levels are quantified through chromogenic substrate reaction, with signal intensity proportional to acetylation.

The determination of hepatic HDAC activity comprised two steps: nuclear protein extraction and HDAC activity determination. The protocol is detailed below: (1) Nuclear proteins were extracted from the liver using the EpiQuik™ Nuclear Extraction Kit (Base Catalog #P-0002; Epigentek, Farmingdale, NY, USA) following the instruction of the manufacturer. (2) HDAC activity was measured using the Epigenase™ HDAC Activity/Inhibition Direct Assay Kit (Colorimetric) (Base Catalog #P-4034; Epigentek, Farmingdale, NY, USA) according to the manufacturer’s protocol. The principle of the assay is that active HDACs bind to acetylated histone substrates coated onto the microplate and deacetylate the substrate. The deacetylated product is detected by a specific antibody, and HDAC activity is quantified by measuring absorbance at 450 nm using a microplate spectrophotometer, with signal intensity correlating to enzymatic activity.

### 2.8. Expression of Key Fatty Acid Oxidation-Related Genes in the Liver

The liver samples stored at −80 °C were thawed and ground into powder in liquid nitrogen. Total RNA was extracted from 50 to 100 mg of the sample using a Trizol reagent (Invitrogen, Carlsbad, CA, USA). RNA quality and integrity were assessed by 1.0% agarose gel electrophoresis, and concentration was determined using a nucleic acid analyzer (Beckman DU-800, Beckman-Coulter, Fullerton, CA, USA). RNA was reverse-transcribed into cDNA using a reverse transcription kit (Takara Bio Inc. Shiga, Japan). The RT-PCR was performed on a real-time PCR instrument (ABI 7900HT) with the following reaction mixture: 5 µL SYBR Premix Ex Taq™ (2×), 0.5 µL each of forward and reverse primers, 1 µL cDNA, and 3 µL DEPC water. Thermal cycling conditions were 95 °C for 1 min for initial denaturation, followed by 40 cycles of 95 °C for 5 s for denaturation and 60 °C for 30 s for annealing. Melting curve analysis was performed from 50 °C to 95 °C with a heating rate of 0.1 °C/s. The relative expression levels were calculated using the 2^−ΔΔCT^ method. The primer sequences are listed in Table S3.

### 2.9. Organ Indices of Piglets at the Birth, Weaning, and Finishing Stages

After slaughter, the heart, liver, lungs, spleen, pancreas, and kidneys of the newborn piglets, weaned piglets, and finishing pigs were weighed. Additionally, the abdominal fat content of the weaned piglets and finishing pigs was measured. The organ indices were then calculated using the following formula: Organ index = (Organ weight/Live body weight) × 100%.

### 2.10. Determination of Carcass Traits and Meat Quality in Finishing Pigs

After slaughter, the finishing pigs were weighed to calculate slaughter yield. The left half of the carcass was used to assess carcass quality, including average backfat thickness and *longissimus dorsi* muscle area (LMA), while the right half (specifically the *longissimus dorsi* muscle between the 13th and 14th ribs) was used to evaluate meat quality parameters: pH at 45 min (pH_45min_), pH at 24 h (pH_24h_), pH difference between pH_45min_ and pH_24h_, meat color (lightness L*, redness a*, and yellowness b*), drip loss, cooking loss, and shear force. Backfat thickness was measured at three points on the left carcass (widest shoulder point [h1], thoracolumbar junction [h2], and lumbosacral junction [h3]), with the average recorded as the final value. For LMA, a cross-section between the 12th and 13th ribs was excised, traced on grid paper, and calculated in cm^2^. pH_45min_ was measured within 45 min post-slaughter using a handheld pH meter (pH-STAR, SFK-Technology, Copenhagen, Denmark), followed by pH_24h_ measurement after 24 h storage at 4 °C. Meat color was assessed at 24 h post-slaughter with a portable colorimeter (CR-300, Minolta Camera, Osaka, Japan). Cooking loss was determined by boiling a 50 g sample to 75 °C core temperature, cooling it in ice slurry, and reweighing after equilibration at 1–5 °C. Drip loss was calculated from 3 × 2 × 1 cm meat pieces suspended at 4 °C for 24 h (Honikel, 1998) [48]. Shear force was measured using a Texture Analyzer (TA.XT. Plus, Stable Micro Systems, Godalming, UK) equipped with a Warner–Bratzler shear device after heating samples to 70 °C in a water bath and chilling overnight at 4 °C (Zhang et al., 2015) [49].

### 2.11. Statistics

The litter served as the observational unit for analyzing growth performance data, whereas the individual pig served as the observational unit for all the other measurements. All the statistical analyses were conducted using two-way ANOVA in SPSS 19.0 (IBM^®^ SPSS^®^ Statistics, New York, NY, USA). When the interaction effects were significant, a one-way ANOVA using a GLM procedure followed by Bonferroni’s post hoc test was performed. The values were presented as mean ± SEM. *p* < 0.05 was considered statistically significant, and 0.05 < *p* < 0.1 was considered to indicate a trend.

## 3. Results

### 3.1. Organ Index of Piglets at Birth and Weaning

As shown in Table 1, inulin supplementation in the gestation diet of sows showed no significant effect on the organ indices of the newborn piglets. However, it significantly elevated the liver index by 13.4% in the weaned piglets (*p* = 0.03) and exhibited an interaction with fat supplementation on the abdominal fat index (*p* = 0.05). Specifically, when dietary fat was supplemented, maternal inulin addition reduced the relative abdominal fat content in weaned piglets.

### 3.2. Growth Performance of Piglets from Weaning to Finishing

As reported in our previous publication [50], inulin supplementation in the gestation diet of sows significantly reduced the weaning weight of the piglets. In this experiment, the piglets in each treatment group maintained the same grouping as their dams throughout the trial (as described in Section 2.1). Consequently, the initial weight of the piglets (weaning weight at day 28 of age) was significantly lower in the inulin-supplemented group (7.88 vs. 7.30 kg; *p* = 0.02). Moreover, the weight-reducing effect of the maternal inulin supplementation on the offspring piglets persisted until day 70 of age (19.23 vs. 18.53; *p* = 0.01), after which no significant differences in body weight were observed among the treatment groups up to day 180 of age (*p* > 0.05). Consistent with the weight change, the piglets in the inulin-supplemented group exhibited significantly lower ADFI compared to the control group up to day 110 of age (*p* < 0.05). However, no significant differences in ADFI were detected from day 111 to 180 of age (*p* > 0.05; Table 2).

### 3.3. Organ Indices and Carcass Traits of Pigs at Finishing Stage

As shown in Table 3, the maternal inulin supplementation during gestation increased the liver index by 6.8% in finishing pigs (*p* = 0.04), with no significant effect on the other organ indices. Furthermore, the inulin supplementation reduced the average backfat thickness by 11.6% (*p* < 0.01), whereas the dietary fat supplementation increased backfat thickness in the finishing pigs (*p* < 0.01).

### 3.4. Meat Quality of Pigs at Finishing Stage

As presented in Table 4, the fat supplementation significantly elevated the pH_dif_ (difference between pH_45min_ and pH_24h_) of *longissimus dorsi* muscle by 26.9% (1.08 vs. 1.37; *p* = 0.04) while exerting no effect on either pH_45min_ or pH_24h_ (*p* > 0.05). Inulin addition, conversely, significantly decreased muscle lightness (L*) by 4.5% (51.59 vs. 49.25; *p* = 0.02), without altering redness (a*) or yellowness (b*) measured 24 h post-slaughter in the finishing pigs.

### 3.5. Hepatic Lipid Deposition in Pigs at Birth and Finishing Stages

**Figure 1:**
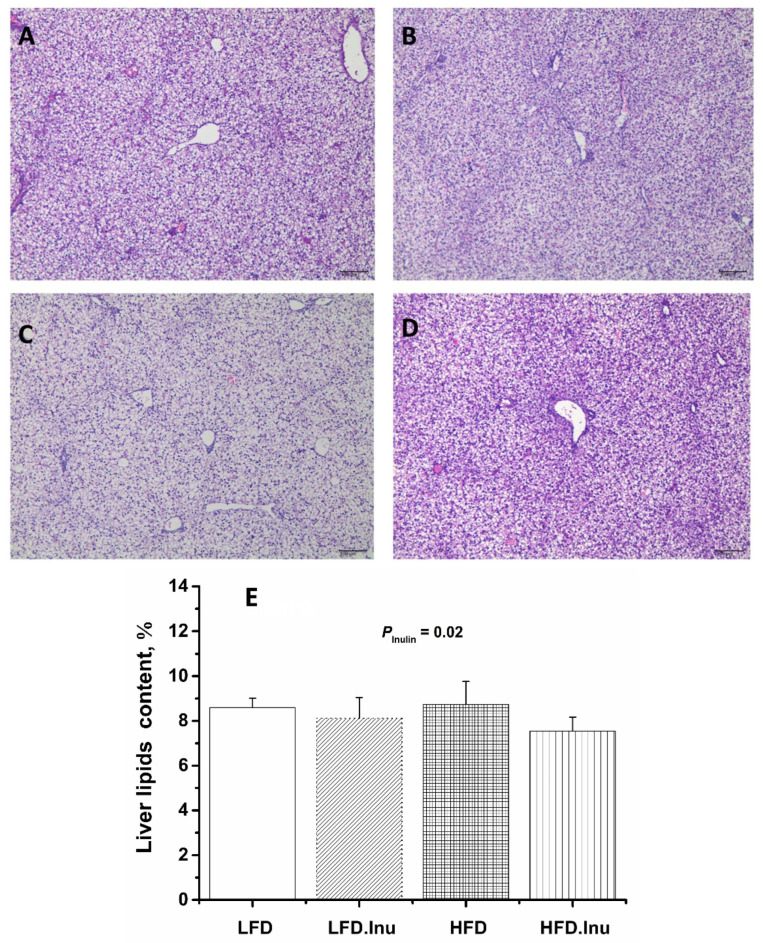
Lipid deposition in the liver of the newborn piglets. (**A**–**D**): H&E staining at magnification of 100×, bar = 100 µm. (**A**) LFD group. (**B**) LFD.Inu group. (**C**) HFD group. (**D**) HFD.Inu group. (**E**) Lipid content in the liver. LFD, low-fat diet; LFD.Inu, low-fat diet with 1.5% inulin; HFD, high-fat diet; HFD.Inu, high-fat diet with 1.5% inulin. The data are expressed as mean ± SEM.

As shown in Figure 1, lipid droplet vacuoles of varying sizes were present in the cytoplasm of the newborn piglet livers across all the treatment groups. However, the lipid droplet vacuoles in the HFD group were particularly severe, with significantly reduced cytoplasmic staining compared to the other three groups, and the nuclei were pushed to the cell periphery by the vacuoles (Figure 1A–D). Additionally, the results of the liver lipid content indicated that inulin supplementation significantly reduced liver lipid content by 9.6% (Figure 1E, *p* = 0.02).

**Figure 2:**
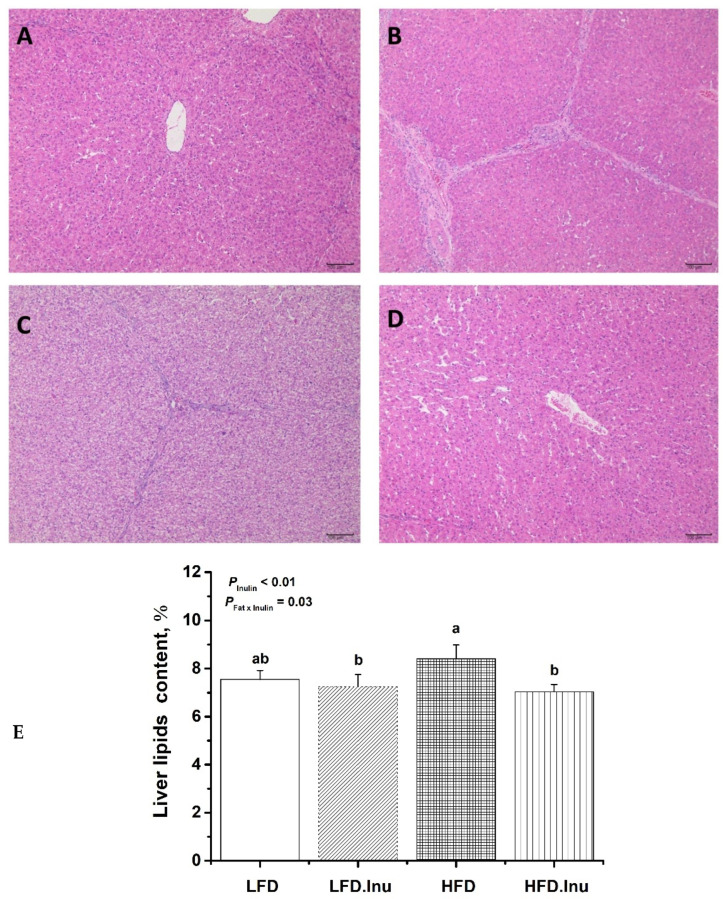
Lipid deposition in the liver of the finishing pigs. (**A**–**D**): H&E staining at magnification of 100×, bar = 100 µm. (**A**) LFD group. (**B**) LFD.Inu group. (**C**) HFD group. (**D**) HFD.Inu group. (**E**) Lipid content in the liver. LFD, low-fat diet; LFD.Inu, low-fat diet with 1.5% inulin; HFD, high-fat diet; HFD.Inu, high-fat diet with 1.5% inulin. The data are expressed as mean ± SEM. Mean values without a common letter are significantly different (*p* < 0.05).

As shown in Figure 2, the cytoplasm of the finishing pigs in the three groups other than the HFD group was uniformly red-stained. In contrast, the liver cords in the HFD group were more disorganized, and lipid droplet vacuoles of varying sizes were visible in the cytoplasm (Figure 2A–D). Furthermore, the results of the liver lipid content indicated that the inulin supplementation significantly reduced liver lipid deposition by 9.9% (*p* < 0.01) and demonstrated a significant interaction with the fat supplementation (*p* = 0.03). Specifically, the reduction in liver lipid deposition was more pronounced when inulin was added in combination with fat supplementation (Figure 2E).

### 3.6. Serum VFA Concentrations in Newborn Piglets

**Figure 3:**
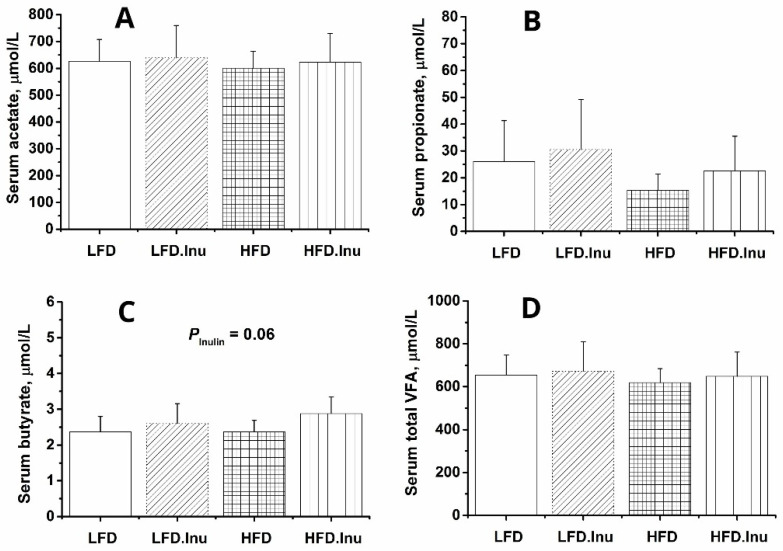
Concentration of serum volatile fatty acids (VFAs) in the newborn piglets: (**A**) acetate; (**B**) propionate; (**C**) butyrate; (**D**) total VFAs. LFD, low-fat diet; LFD.Inu, low-fat diet with 1.5% inulin; HFD, high-fat diet; HFD.Inu, high-fat diet with 1.5% inulin. The data are expressed as mean ± SEM.

As shown in Figure 3, the inulin supplementation during gestation did not significantly affect the serum concentrations of acetate, propionate, and total VFAs in the newborn piglets. However, the inulin supplementation increased serum butyrate concentration by 15.6% in the newborn piglets (Figure 3C, *p* = 0.06).

### 3.7. HDAC Activity and Histone Acetylation in the Liver of Newborn Piglets

**Figure 4:**
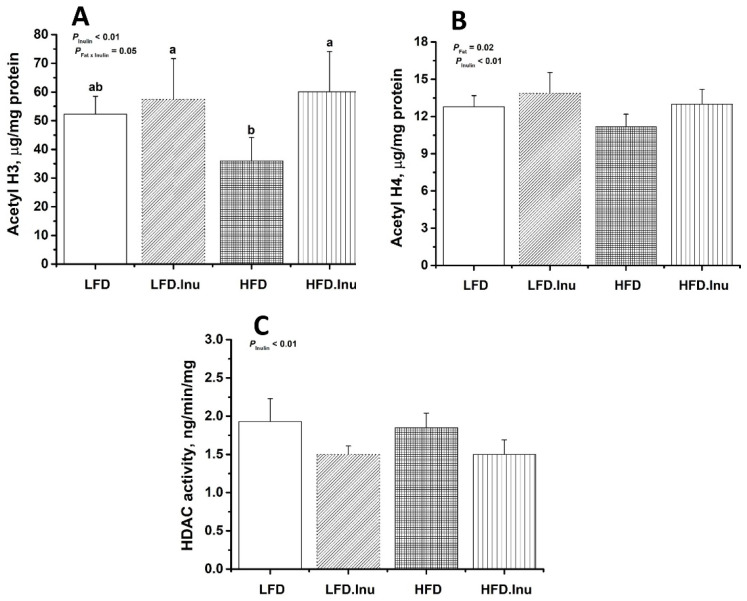
Histone deacetylase (HDAC) activity and the acetylation of histones H3 and H4 in the liver of the newborn piglets: (**A**) Acetyl H3; (**B**) Acetyl H4; (**C**) HDAC activity. The data are expressed as mean ± SEM. LFD, low-fat diet; LFD.Inu, low-fat diet with 1.5% inulin; HFD, high-fat diet; HFD.Inu, high-fat diet with 1.5% inulin. Mean values without a common letter are significantly different (*p* < 0.05).

As shown in Figure 4, the inulin supplementation during sow gestation significantly increased the levels of acetylated histone H3 (Figure 4A) and H4 (Figure 4B) in the liver of the newborn piglets (*p* < 0.01). Additionally, a significant interaction between the fat and inulin supplementation was observed for the acetylated H3 levels (Figure 4A, *p* = 0.05), whereas the fat supplementation significantly reduced the acetylated H4 levels (Figure 4B, *p* = 0.02). Among the four groups, the piglets in the HFD group exhibited the lowest levels of acetylated H3 (Figure 4A). Furthermore, the inulin supplementation significantly reduced hepatic HDAC activity (Figure 4C, *p* < 0.01).

### 3.8. Expression of Key Genes Involved in Hepatic Fatty Acid Oxidation in Pigs at Birth and Finishing Stage

**Figure 5:**
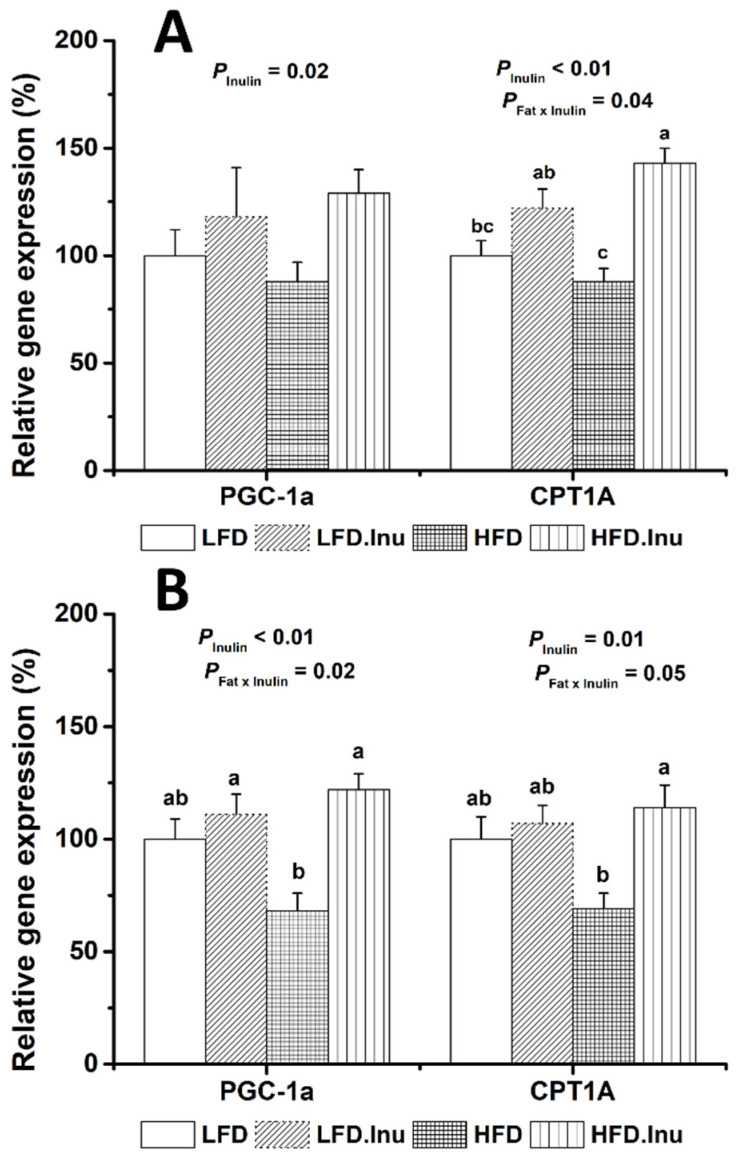
Expression of the key genes involved in fatty acid oxidation in the liver of the newborn piglets (**A**) and finishing pigs (**B**). The data are expressed as mean ± SEM. Mean values without a common letter are significantly different (*p* < 0.05). LFD, low-fat diet; LFD.Inu, low-fat diet with 1.5% inulin; HFD, high-fat diet; HFD.Inu, high-fat diet with 1.5% inulin.

As shown in Figure 5A, the inulin supplementation during sow gestation significantly increased the hepatic expression of *PGC-1α* (*p* = 0.02) and *CPT1A* (*p* < 0.01) in the newborn piglets, and also demonstrated a significant interaction with the fat supplementation on *CPT1A* expression (*p* = 0.04), where the effect was more pronounced when combined with the fat supplementation.

As shown in Figure 5B, the inulin supplementation during sow gestation also significantly increased the hepatic expression of *PGC-1α* (*p* < 0.01) and *CPT1A* (*p* = 0.01) in the finishing pigs. Moreover, both genes displayed significant inulin × fat supplementation interactions (*p* = 0.02 and *p* = 0.05, respectively), where the effect was more pronounced when combined with the fat supplementation.

## 4. Discussion

The SCFAs, metabolic derivatives of dietary fiber fermentation, have been shown to exert their regulatory effects on gene expression through epigenetic modifications [20]. Notably, butyrate, one of the end products of microbial fermentation of dietary fiber in the hindgut, is a potent HDAC inhibitor with the smallest molecular weight, capable of activating gene expression [21]. We hypothesize that maternally derived SCFAs from dietary fiber fermentation undergo placental transfer to induce fetal epigenetic reprogramming through histone deacetylation, thereby programming postnatal growth and metabolic trajectories. Our experimental data revealed a trend toward increased serum butyrate levels in newborn piglets from inulin-supplemented sows, providing first-tier validation of our proposed mechanistic framework.

The liver plays an important role in fat metabolism, acting as the central hub for fatty acid synthesis and lipid accumulation [51] while also acting as a target of maternal nutritional programming [52]. Increased liver weight likely reflects greater metabolic activity or may indicate increased mRNA expression of lipid-metabolizing enzymes in hepatocytes [53]. Notably, our data revealed that dietary inulin supplementation significantly elevated the liver index in both weaned piglets and finishing pigs, indicative of a potential sustained state of hepatic metabolic activity. The maternal inulin supplementation during gestation reduced hepatic lipid deposition in newborn piglets, a phenomenon potentially linked to butyrate-mediated mechanisms. Butyrate has been shown to ameliorate obesity in diet-induced obese mice, improve insulin sensitivity, and alleviate lipid disorders [25,26,54,55]. As a critical metabolic process, fatty acid oxidation serves as an important pathway for cellular energy production [56]. The beneficial effects of butyrate are primarily attributed to its ability to enhance energy expenditure and promote fatty acid β-oxidation [23,57]. To elucidate this mechanism, we investigated the expression of two key genes involved in hepatic fatty acid *β*-oxidation: peroxisome proliferator-activated receptor γ coactivator-1α (*PGC-1α*) and carnitine palmitoyltransferase-1A (*CPT1A*). *PGC-1α*, a nuclear transcription coactivator abundantly expressed in the liver, plays a central role in regulating fatty acid oxidation and hepatic gluconeogenesis [58]. Notably, sodium butyrate supplementation has been consistently shown to upregulate *PGC-1α* expression in diverse tissues or cells, driving phenotypic improvements such as weight reduction in obesity [59], attenuation of hepatic steatosis in high-fat fed rats [60], and enhanced steroidogenesis in ovarian granulosa cells [61]. This regulatory effect of butyrate is mechanistically tied to its inhibition of HDAC activity, which elevates histone acetylation levels and facilitates gene transcription [60,61]. Supporting this, Ye et al. (2024) [61] found that butyrate treatment (0.4–1.6 mmol/L) significantly suppressed nuclear HDAC activity and increased acetylated histone H3K9 levels in human ovarian granulosa cells. CPT1A, the rate-limiting enzyme in mitochondrial fatty acid β-oxidation, governs the transport of fatty acids into mitochondria. A reduction in *CPT1A* levels impairs fatty acid shuttling, leading to cytoplasmic lipid accumulation and exacerbated lipid deposition [62]. Reinforcing our findings, Li et al. (2012) [23] reported that sodium butyrate administration in diet-induced obese mice elevated hepatic *CPT1A* expression by over twofold, accompanied by a marked rise in serum ketone bodies, a hallmark of enhanced β-oxidation. Furthermore, metabolic calorimetry revealed a reduced respiratory exchange ratio (RER) in these mice, indicating a systemic shift toward fatty acid utilization [23]. In our study, the upregulation of both PGC-1α and CPT1A in the livers of newborns and finishing pigs provides a mechanistic basis for the observed reduction in hepatic lipid deposition following maternal inulin supplementation.

Butyrate exerts its effects through multiple mechanisms, most of which are related to its inhibition of class I and II HDACs activity, thereby elevating histone acetylation levels and activating gene expression [21,63]. To validate this mechanistic link, we examined hepatic HDAC activity and acetylated histone H3 and H4 levels in newborn piglets. Our results revealed that inulin supplementation during gestation significantly suppressed hepatic HDAC activity concomitantly with the increased acetylation of histones H3 and H4. Collectively, these findings support the hypothesis that maternally derived butyrate (produced from gestational inulin fermentation) is transferred to the fetus, where it attenuates hepatic HDAC activity and enhances histone acetylation. This epigenetic reprogramming likely activates the transcription of fatty acid oxidation genes (e.g., *PGC-1α* and *CPT1A*), ultimately leading to a reduction in hepatic lipid deposition in offspring.

Gene-regulated epigenetic mechanisms are recognized to possess mitotic stability, enabling persistent transcriptional activation [64,65]. To assess the long-term implications of this phenomenon, we evaluated the effects of maternal inulin supplementation on offspring growth performance, carcass traits, and hepatic lipid metabolism across developmental stages (from birth to finishing). Although the maternal inulin treatment did not alter most carcass or meat quality parameters in the finishing pigs, notably, offspring from the inulin-supplemented group showed reduced abdominal fat and lower average backfat thickness at both the weaning and finishing stages. Furthermore, the hepatic expression of the fatty acid oxidation genes *PGC-1α* and *CPT1A* in the finishing pigs from the maternal inulin group was significantly upregulated (mirroring the neonatal pattern), concomitant with attenuated liver lipid deposition. Notably, we observed an interesting developmental pattern: the offspring from the inulin-supplemented group exhibited significantly reduced ADFI from day 28 to 110 of age, leading to a noticeable retardation in weight gain. However, after day 110 of age, these offspring displayed progressive increases in feed consumption, ultimately achieving body weights comparable to the control groups. Whether these phenotypic changes presage the emergence of compensatory growth capacity in the offspring from the inulin-supplemented group during later growth-finishing stages remains empirical confirmation through further investigation.

## 5. Conclusions

Our findings demonstrated that maternal inulin supplementation during gestation mitigates offspring hepatic lipid deposition through butyrate-mediated epigenetic regulation, where microbial-derived butyrate from inulin fermentation inhibits HDAC activity, enhances the acetylation of histones H3 and H4, and upregulates the expression of fatty acid β-oxidation-related genes. These stable acetylation-driven epigenetic modifications persisted even into the finishing stage of the offspring, yet exhibited no significant impact on most carcass traits or meat quality parameters. This study provides novel mechanistic insights into how maternal dietary fiber nutrition programs offspring development through epigenetic reprogramming.

## Figures and Tables

**Table 1:** Effect of inulin supplementation to gestating sows on organ indices of offspring pigs at birth and weaning.

	Maternal Treatment				SEM	*p*-Value		
	LFD	LFD.Inu	HFD	HFD.Inu		Fat	Inulin	Fat × Inulin
Piglet BW, kg								
At birth	1.25	1.24	1.32	1.27	0.01	0.06	0.22	0.46
At weaning	7.56	7.33	7.93	7.28	0.08	0.36	0.02	0.22
Organ index of newborn piglets, %								
Heart	0.72	0.68	0.63	0.70	0.02	0.41	0.76	0.27
Liver	2.67	2.84	2.59	2.73	0.06	0.46	0.23	0.92
Spleen	0.08	0.09	0.10	0.08	0.003	0.31	0.41	0.22
Lung	1.31	1.43	1.43	1.34	0.04	0.90	0.87	0.23
Kidney	0.63	0.65	0.66	0.61	0.02	0.86	0.71	0.43
Pancreas	0.08	0.09	0.08	0.07	0.002	0.38	0.56	0.06
Organ index of weaning piglets, %								
Heart	0.51	0.49	0.51	0.48	0.01	0.86	0.26	0.72
Liver	2.21	2.66	2.42	2.58	0.06	0.62	0.03	0.28
Spleen	0.24	0.28	0.19	0.33	0.02	0.99	0.07	0.31
Lung	1.12	1.36	1.23	1.23	0.06	0.98	0.32	0.32
Kidney	0.48	0.51	0.47	0.53	0.02	0.79	0.15	0.65
Pancreas	0.13	0.11	0.10	0.12	0.006	0.46	0.85	0.25
Abdominal fat	0.30	0.31	0.37	0.27	0.01	0.54	0.09	0.05

LFD, low-fat diet; LFD.Inu, low-fat diet with 1.5% inulin; HFD, high-fat diet; HFD.Inu, high-fat diet with 1.5% inulin. The data are expressed as mean ± pooled SEM. Within a row, mean values without a common letter are significantly different (*p* < 0.05).

**Table 2:** Effect of inulin supplementation to gestating sows on growth performance of offspring pigs from weaning to finishing period.

	Maternal Treatment				SEM	*p*-Value		
	LFD	LFD.Inu	HFD	HFD.Inu		Fat	Inulin	Fat × Inulin
Average pig weight, kg								
Day 28	7.64	7.31	8.12	7.29	0.11	0.31	0.02	0.27
Day 70	19.55	18.91	20.18	16.89	0.35	0.33	0.01	0.07
Day 110	47.37	47.68	46.19	41.95	0.67	0.02	0.16	0.11
Day 150	78.88	74.93	75.88	74.06	1.09	0.39	0.20	0.63
Day 180	99.76	96.76	98.24	95.91	0.93	0.53	0.17	0.86
Average pig weight gain, kg	92.12	89.45	90.12	88.62	0.89	0.44	0.26	0.75
ADFI, g/d								
Day 28 to 70	492	456	480	408	9	0.12	0.01	0.34
Day 71 to 110	1430	1184	1306	1164	32	0.26	<0.01	0.41
Day 111 to 150	2152	1952	1970	1966	43	0.35	0.26	0.28
Day 151 to 180	2380	2470	2354	2358	85	0.69	0.79	0.80
Day 28 to 180	1550	1440	1458	1402	28	0.26	0.16	0.63

The litter served as the experimental unit for analyzing growth performance data. LFD, low-fat diet; LFD.Inu, low-fat diet with 1.5% inulin; HFD, high-fat diet; HFD.Inu, high-fat diet with 1.5% inulin. The data are expressed as mean ± pooled SEM. Within a row, mean values without a common letter are significantly different (*p* < 0.05).

**Table 3:** Effect of inulin supplementation to gestating sows on organ indices and carcass traits of offspring pigs at slaughter.

	Maternal Treatment				SEM	*p*-Value		
	LFD	LFD.Inu	HFD	HFD.Inu		Fat	Inulin	Fat × Inulin
BW, kg	99.44	98.60	100.68	97.56	0.50	0.92	0.07	0.27
Organ index, %								
Heart	0.32	0.34	0.29	0.32	0.006	0.12	0.12	0.70
Liver	1.42	1.58	1.49	1.53	0.02	0.79	0.01	0.10
Spleen	0.13	0.16	0.15	0.14	0.004	0.90	0.18	0.08
Lung	0.67 ^b^	1.07 ^a^	1.04 ^ab^	0.74 ^ab^	0.05	0.80	0.61	<0.01
Kidney	0.31	0.33	0.30	0.30	0.005	0.04	0.35	0.45
Pancreas	0.13	0.12	0.11	0.12	0.004	0.47	1.00	0.47
Abdominal fat	0.86	0.76	0.86	0.77	0.04	0.99	0.27	0.92
Dressing Percentage, %	71.24	71.70	72.87	72.43	0.39	0.15	0.99	0.57
Average backfat depth, cm	1.80	1.49	1.99	1.87	0.03	<0.01	<0.01	0.14
LMA ^1^, cm	63.29	65.73	61.76	64.07	1.07	0.31	0.14	0.97

^1^ LMA, area of the *longissimus dorsi* muscle between the 12th and 13th ribs of the left half of the carcass. LFD, low-fat diet; LFD.Inu, low-fat diet with 1.5% inulin; HFD, high-fat diet; HFD.Inu, high-fat diet with 1.5% inulin. The data are expressed as mean ± pooled SEM. Within a row, mean values without a common letter are significantly different (*p* < 0.05).

**Table 4:** Effect of inulin supplementation to gestating sows on meat quality of offspring pigs at slaughter.

	Maternal Treatment				SEM	*p*-Value		
	LFD	LFD.Inu	HFD	HFD.Inu		Fat	Inulin	Fat × Inulin
pH_45min_	6.47	6.66	6.53	6.77	0.05	0.47	0.06	0.80
pH_24h_	5.39	5.59	5.22	5.34	0.05	0.07	0.13	0.70
pH_dif_	1.09	1.07	1.31	1.43	0.07	0.04	0.70	0.60
Meat color_24h_								
L*	50.76	49.38	52.41	49.12	0.47	0.46	0.02	0.32
a*	6.14	6.39	6.19	6.37	0.10	0.94	0.29	0.87
b*	6.55	6.20	6.49	6.34	0.19	0.93	0.52	0.81
Cooking loss, %	31.86	29.87	32.47	29.94	0.91	0.85	0.23	0.89
Drip loss, %	2.74	2.53	2.53	2.46	0.06	0.27	0.26	0.58
Shear force, N	4.75	4.83	4.76	4.78	0.04	0.82	0.49	0.71

pH_dif_, difference between pH_45min_ and pH_24_. LFD, low-fat diet; LFD.Inu, low-fat diet with 1.5% inulin; HFD, high-fat diet; HFD.Inu, high-fat diet with 1.5% inulin. The data are expressed as mean ± SEM. Within a row, mean values without a common letter are significantly different (*p* < 0.05).

## Data Availability

The original contributions presented in the study are included in the article/Supplementary Materials, further inquiries can be directed to the corresponding authors.

## Supplementary Materials

The following supporting information can be downloaded at https://www.mdpi.com/article/10.3390/foods14081314/s1, Table S1: Ingredient and nutrient composition of gestational diets (as-fed basis); Table S2: Composition and nutrient levels of diets at different stages (as-fed basis); Table S3: Primer sequences of target and reference genes.
