# Supplementary material for: Gestational Inulin Supplementation in Low-/High-Fat Sow Diets: Effects on Growth Performance, Lipid Metabolism, and Meat Quality of Offspring Pigs
Source: Foods. 2025 Apr 10;14(8):1314. doi: 10.3390/foods14081314 (PMC12027208; doi:10.3390/foods14081314)
Supplement: Supplementary file 1 [file foods-14-01314-s001.zip › foods-3519102-supplementary.pdf]

Table S1 Ingredient and nutrient composition of gestational diets (as-fed basis)

Item	Low fat		High fat	
	0% inulin (LFD)	1.5% inulin (LFD.Inu)	0% inulin (HFD)	1.5% inulin (HFD.Inu)
Ingredient, %				
Corn	55.83	55.83	55.83	55.83
Soybean meal,	15.12	15.12	15.12	15.12
Wheat bran	18.50	18.50	18.50	18.50
Soybean oil	-	-	5.00	5.00
Maize starch	6.50	5.00	1.50	-
Inulin[1]	-	1.50	-	1.50
L-Lysine (98.5%)	0.04	0.04	0.04	0.04
DL-Methionine (99%)	0.05	0.05	0.05	0.05
L-Threonine (98.5%)	0.06	0.06	0.06	0.06
Calcium carbonate	1.11	1.11	1.11	1.11
Monocalcium phosphate	1.69	1.69	1.69	1.69
Choline	0.15	0.15	0.15	0.15
Salt	0.40	0.40	0.40	0.40
Vitamin premix[2]	0.05	0.05	0.05	0.05
Trace mineral premix[3]	0.50	0.50	0.50	0.50
Nutrient composition				
Digestible energy, MJ/kg	12.85	12.60	13.86	13.60
Crude protein, %	13.80	13.79	13.78	13.78
Crude fat, %	2.99	2.99	7.88	7.88
Crude fiber, %	3.04	4.39	3.04	4.39
Lysine, %	0.66	0.66	0.66	0.66
Methionine +	0.41	0.41	0.41	0.41
Threonine, %	0.47	0.47	0.47	0.47
Tryptophan, %	0.13	0.13	0.13	0.13
Ca, %	0.90	0.90	0.90	0.90
Available P, %	0.45	0.45	0.45	0.45

[1]Inulin was purchased from BENEO-Orafti (Orafti, Belgium) with a purity exceeding 90% and an average degree of polymerization (DP) of 10-12.

[2]Provided per kg of diet: 4000 IU vitamin A; 800 IU vitamin D3; 441 IU vitamin E; 0.5 mg vitamin K; 1.0 mg vitamin B1; 3.75 mg vitamin B2; 1.0 mg vitamin B6; 15 μg vitamin B12; 10 mg niacin; 12 mg pantothenic acid; 1.3 mg folic acid; 200 μg D-biotin.

[3]Provided per kg of diet: 165 mg Fe; 16 mg Cu; 165 mg Zn; 30 mg Mn; 0.3 mg Se; 0.3 mg I.

Table S2 Composition and nutrient levels of diets at different stages (as-fed basis)

Ingredients (%)	d 28-42	d 42-70	d 70-110	d 110-150	d 150-180
Corn	12.57	42.57	68.70	72.30	70.96
Soybean meal	-	-	25.20	22.60	19.62
Extruded rice	15.00	15.00	-	-	-
Extruded corn	15.00	-	-	-	-
Wheat bran	-	-	-	-	4.00
Fish meal	5.00	3.00	-	-	-
Extruded soybean	9.00	5.00	-	-	-
Plasma protein	4.00	-	-	-	-
Fat powder	2.00	1.00	-	-	-
Dehulled soybean meal	8.00	15.00	-	-	-
Soybean oil	-	-	2.50	2.10	2.80
Soy protein	-	1.00	-	-	-
Fermented soybean meal	5.00	3.00	-	-	-
Whey powder	15.00	8.00	-	-	-
Sucrose	3.00	2.00	-	-	-
Glucose	2.00	0.00	-	-	-
Limestone	0.50	0.50	0.90	0.89	0.87
Dicalcium phosphate	0.80	1.00	1.20	0.78	0.60
Choline chloride	0.10	0.10	0.10	0.10	0.10
Salt	0.15	0.30	0.30	0.30	0.30
L-Lysine HCl (98%)	0.50	0.40	0.36	0.31	0.19
D,L-Methionine (99%)	0.20	0.10	0.11	0.06	0.03
L-Threonine (98.5%)	0.25	0.10	0.12	0.05	0.03
L-Tryptophan (98.5%)	0.05	0.02	0.01	0.01	-
Vitamin premix[1]	0.05	0.05	0.03	0.03	0.03
Mineral premix[2]	1.83	1.86	0.47	0.47	0.47
Total	100.00	100.00	100.00	100.00	100.00
Calculated nutrient composition					
DE (Mcal/kg)	3.45	3.40	3.40	3.40	3.40
CP (%)	20.00	18.00	17.00	16.00	15.00
SID-Lys (%)	1.46	1.35	1.00	0.90	0.75
SID-Met (%)	0.42	0.37	0.33	0.33	0.23
SID-Trp (%)	0.26	0.25	0.17	0.15	0.46
SID-Thr (%)	1.01	0.95	0.60	0.52	0.13
Ca (%)	0.75	0.75	0.70	0.60	0.55
Total P (%)	0.60	0.60	0.55	0.47	0.45
Available P (%)	0.41	0.41	0.34	0.27	0.24

[1]Provided per kg of diet for d 28-42 stage: vitamin A 12000 IU, vitamin E 80 IU, vitamin D_3 3200 IU, vitamin K 25 mg, vitamin B_1 25 mg, vitamin B_2 65 mg, vitamin B_6 5 mg, vitamin B_{12} 0.5 mg, niacin 45 mg, pantothenic acid 20 mg, folic acid 15 mg, biotin 0.15 mg. Provided per kg of diet for d 42-70 stage: vitamin A 12000 IU, vitamin E 80 IU, vitamin D3 3200 IU, vitamin K 25 mg, vitamin B_1 25 mg, vitamin B_2 65 mg, vitamin B_6 5 mg, vitamin B_{12} 0.5 mg, niacin 45 mg, pantothenic acid 20 mg, folic acid 15 mg, biotin 0.15 mg. Provided per kg of diet for d 70-110, d 110-150, and d 150-180 stage: vitamin A 15750 IU, vitamin E 26.2 mg, vitamin D_3 3500 IU, vitamin K 3.5 mg, vitamin B_1 3.5 mg, vitamin B_2 8.8 mg, vitamin B_{12} 26.2 μg, vitamin B_6 17.5 mg, biotin 0.14 mg, folic acid 1.7 mg, pantothenic acid 8 mg, niacin 35 mg.

[2]Provided per kg of diet for d 28-42 and 42-70 stage: Fe 150 mg, Cu 125 mg, Zn 150 mg, Mn 30 mg, I 0.3 mg, Se 0.3mg. Provided per kg of diet for d 70-110, d 110-150, and d 150-180 stage: Fe 150 mg, Cu 20 mg, Zn 150 mg, Mn 20 mg, I 0.3 mg, Se 0.3 mg.

Table S3 Primer sequences of target and reference genes

Gene name	Primer sequence （5`→3`）	GenBank No.
PGC-1α	GATGTGTCGCCTTCTTGTTC<br>CATCCTTTGGGGTCTTTGAG	NM_213963
CPT1A	TCACAAGCGAATTTGAGTGC<br>AAATTCAGACCGCAGTTTCG	AF288789
β-actin	CCAGCACGATGAAGATCAAGA<br>AATGCAACTAACAGTCCGCCTA	AY550069.1
